# Gas Plasma Pre-treatment Increases Antibiotic Sensitivity and Persister Eradication in Methicillin-Resistant *Staphylococcus aureus*

**DOI:** 10.3389/fmicb.2018.00537

**Published:** 2018-03-23

**Authors:** Li Guo, Ruobing Xu, Yiming Zhao, Dingxin Liu, Zhijie Liu, Xiaohua Wang, Hailan Chen, Michael G. Kong

**Affiliations:** ^1^State Key Laboratory of Electrical Insulation and Power Equipment, Center for Plasma Biomedicine, Xi'an Jiaotong University, Xi'an, China; ^2^School of Life Science and Technology, Xi'an Jiaotong University, Xi'an, China; ^3^Frank Reidy Center for Bioelectrics, Old Dominion University, Norfolk, VA, United States; ^4^Department of Electrical and Computer Engineering, Old Dominion University, Norfolk, VA, United States

**Keywords:** cold atmospheric-pressure plasma, antibiotics resistance, methicillin-resistant *Staphylococcus aureus*, reactive oxygen species, reactive nitrogen species

## Abstract

Methicillin-resistant *Staphylococcus aureus* (MRSA) is a major cause of serious nosocomial infections, and recurrent MRSA infections primarily result from the survival of persister cells after antibiotic treatment. Gas plasma, a novel source of ROS (reactive oxygen species) and RNS (reactive nitrogen species) generation, not only inactivates pathogenic microbes but also restore the sensitivity of MRSA to antibiotics. This study further found that sublethal treatment of MRSA with both plasma and plasma-activated saline increased the antibiotic sensitivity and promoted the eradication of persister cells by tetracycline, gentamycin, clindamycin, chloramphenicol, ciprofloxacin, rifampicin, and vancomycin. The short-lived ROS and RNS generated by plasma played a primary role in the process and induced the increase of many species of ROS and RNS in MRSA cells. Thus, our data indicated that the plasma treatment could promote the effects of many different classes of antibiotics and act as an antibiotic sensitizer for the treatment of antibiotic-resistant bacteria involved in infectious diseases.

## Introduction

Antibiotics are the primary treatment for infectious bacterial diseases (Li et al., 2017). Antibiotic resistance typically emerges several years after the development and use of a new antibiotic, usually within an average of 8 years after introduction (Schmieder and Edwards, 2012). Furthermore, infections with multidrug-resistant bacteria are occurring more frequently, and few or no drugs are available to combat them (Boucher et al., 2009; Wright, 2016; Gonzalez-Bello, 2017). One of the major multidrug-resistant bacteria is methicillin-resistant *Staphylococcus aureus* (MRSA), which is recognized as a leading cause of nosocomial infections (Shahsavan et al., 2012; Emaneini et al., 2017). Persisters are a small non-growing population of bacteria which could also escape from different antibiotics (Levin and Rozen, 2006). Persisters extend the duration of antibiotic treatment, cause the recurrence of infectious diseases, and the generation and ascent of antibiotic resistance (Levin and Rozen, 2006). Increased antibiotic resistance and the shortage of new antibiotics threaten global public health (Boucher et al., 2009; Dwyer et al., 2014). Therefore, strategies to combat antibiotic resistance, such as the development of new antibiotics or prolonging the lifespan of current antibiotics, are in high demand (Melander and Melander, 2017).

One strategy that has been explored recently is developing approaches that induce bacterial killing via the same mechanism as existing antibiotics. Antibiotics with different targets have been proposed to share a common mechanism of bactericidal activity—enhancing intracellular reactive oxygen species (ROS) in bacterial cells (Kohanski et al., 2007, 2010). Although ROS could be developed as antimicrobials to treat infectious diseases, treatment with these highly reactive molecules is problematic because ROS react non-selectively with such a range of critical, macromolecular targets, which could lead to “off-target” effects (Dharmaraja, 2017). Given that oxidative stress is also associated with antibiotic treatment, low levels of ROS could be used as an adjuvant to potentiate the antibacterial activity of commercial antibiotics (Brynildsen et al., 2013; Morones-Ramirez et al., 2013; Shen et al., 2016). A previous study showed that ^•^OH induced in Ag^+^-treated bacteria potentiated the bactericidal activity of antibiotics against bacterial persisters and biofilms (Morones-Ramirez et al., 2013). Furthermore, fosfomycin, which acts by generating ${\text{O}}_{2}^{•-}$, was used to successfully treat MRSA infections when combined with many commercial antibiotics (Shen et al., 2016). These strategies relied on the addition of inorganic or organic chemicals, which would bring residues after the treatment.

Cold atmospheric-pressure plasma (referred to as “plasma”) generates many reactive oxygen and nitrogen species (ROS and RNS), such as H_2_O_2_, ^1^O_2_, O_3_, ^•^NO, and ^•^OH as well as electrons, ions, and photons. This form of plasma is atmospheric-pressure and near room temperature, thus it treats cells and tissues without thermal damage, making it attractive for a range of biomedical applications, such as bacteria inactivation (Fridman et al., 2008; Moreau et al., 2008; Kong et al., 2009; Kang et al., 2014). The FDA has authorized the use of at least three gas plasma-based products using “plasma biomedicine” technology, such as blood coagulation. In addition, there have been several phase-II clinical trials for plasma-based therapies, such as chronic wound healing, which is the promising applications of plasma in medicine (Isbary et al., 2010, 2012; Heinlin et al., 2013). In the treatment of wounds, the reactive species of plasma could not only kill the microorganisms in the infectious wounds and burns but also increase proliferation of fibroblasts and other cells (Lloyd et al., 2010). Bayliss et al. (2013) found that treating MRSA with sublethal doses of plasma restored the sensitivity of MRSA to trimethoprim, kanamycin, and oxacillin, but the utility of plasma treatment with other types of antibiotics was not studied.

In the present study, in order to further our understanding of the effect of plasma treatment on antibiotic sensitivity, MRSA was treated with both plasma and plasma-activated saline prior to exposure to multiple antibiotics. Sublethal treatment of MRSA with plasma increased the sensitivity of MRSA to seven antibiotics whilst also reducing the numbers of persisters. These results support the use of plasma as an antibiotic sensitizer for the treatment of antibiotic-resistant bacteria involved in infectious diseases.

## Materials and methods

### Plasma device and plasma treatments

**Figure 1:**
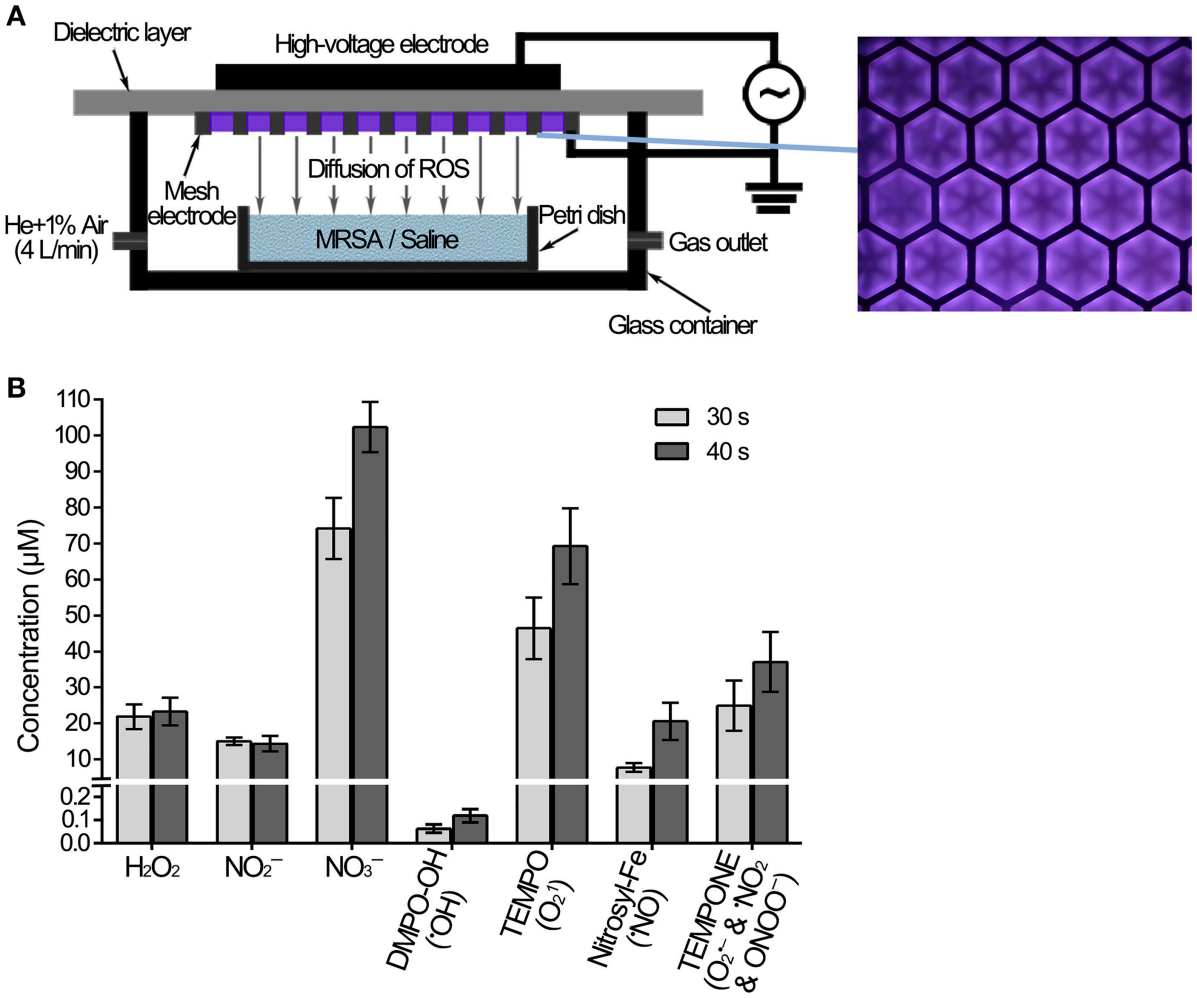
Measurement of the ROS and RNS diffused into the saline treated with plasma. **(A)** Diagram of MRSA suspension or saline treated with plasma. **(B)** The concentrations of ROS, RNS and spin trap adducts diffused into the saline treated with plasma for 30 and 40 s.

As shown in Figure 1A, the surface discharge structure of the plasma consisted of a high-voltage plane electrode, a liquid-facing grounded mesh electrode of a hexagonal shape and a dielectric layer (made of polytetrafluoroethylene) sandwiched between the two electrodes. The surface plasma was generated when a sinusoidal high voltage was applied and the discharge power density was 0.2 W/cm^2^ with good mesh-to-mesh homogeneity (showed in the front view of the plasmas in Figure 1A). One milliliter MRSA suspension or saline in a Petri dish (diameter 35 mm) with the depth of 1 mm was placed under the plasma, whereas the air gap between the plasma and the liquid surface was about 8 mm. The gas plasma system was housed in a sealed organic glass box with a gas flow of helium and 1% artificial air (79% N_2_ + 21% O_2_) at a constant rate of 4 L/min. The artificial air was used as the source of ROS and RNS, while the helium was used to enhance the production efficiency of those species as well as their fluxes on the treated samples via diffusion.

### Measurement of ROS and RNS generated by the plasma

The concentrations of H_2_O_2_ and ${\text{NO}}_{2}^{-}$/${\text{NO}}_{3}^{-}$ in 0.9% NaCl were measured using hydrogen peroxide/peroxidase assay kit (Thermo Fisher Scientific) and nitrite/nitrate colorimetric assay kit (Cayman), respectively. ^•^OH, ^1^O_2_, ^•^NO, ${\text{O}}_{2}^{•-}$, ^•^NO_2_, and ONOO^−^ were measured using an electron spin-resonance (ESR) spectroscopy (Bruker) together with relevant spin traps. The latter include: 100 mM DMPO (5,5-Dimethyl-1-pyrroline N-oxide, Dojindo), 5 mM MGD (N-(Dithiocarbamoyl)-N-methyl-D-glucamine, Dojindo), 10 mM TEMP (2,2,6,6-Tetramethylpiperidine, TCI), and 10 mM TEMPONE-H (1-Hydroxy-2,2,6,6-tetramethyl-4-oxo-piperidine, Enzo).

### Treatment of *Staphylococcus aureus* with plasma or plasma-activated saline

A single *S. aureus* ATCC33591 colony was grown in 4 ml of Mueller-Hinton (MH) broth (Oxoid) at 225 r.p.m. at 37°C overnight, and the resulting overnight culture was diluted 1:100 in MH and incubated at 37°C at 225 r.p.m. until an OD_600_ of 0.6 was reached. The bacterial cells were collected by centrifugation, washed once with saline (0.9% NaCl) and resuspended in saline at an OD_600_ of 2.0. *S. aureus* suspensions were treated with plasma directly, or the suspensions were centrifuged and resuspended in plasma-activated saline (saline treated with plasma for the indicated time), saline with 250 μM H_2_O_2_ + 125 μM ${\text{NO}}_{2}^{-}$ + 375 μM ${\text{NO}}_{3}^{-}$, or saline with 500 μM H_2_O_2_ + 250 μM ${\text{NO}}_{2}^{-}$ + 750 μM ${\text{NO}}_{3}^{-}$ and then incubated for 30 min at room temperature. Samples were serially diluted with 0.9% NaCl, and 10 μl of each dilution was spotted onto MH agar plates and incubated overnight at 37°C and the numbers of surviving bacteria were determined by counting the resulting CFUs.

### Determination of antibiotic sensitivity

The *S. aureus* suspensions prepared as described above were either untreated, treated with plasma directly for 30 or 40 s, or centrifuged and resuspended in plasma-activated saline (saline treated by plasma for 30 or 40 s), followed by incubation for 30 min at room temperature. Untreated *S. aureu*s, or cells treated with either plasma or plasma-activated saline (100 μl), were plated on MH agar plates. Next, antibiotic susceptibility tests were performed using Etest paper (Biomerieux) for tetracycline (0.016–256 μg/ml), gentamycin (0.016–256 μg/ml), clindamycin (0.016–256 μg/ml), chloramphenicol (0.016–256 μg/ml), ciprofloxacin (0.02–32 μg/ml), rifampicin (0.02–32 μg/ml), and vancomycin (0.016–256 μg/ml) or the Kirby-Bauer method for clindamycin (250, 500, 1,000, and 2,000 μg). Then the plates were cultured overnight at 37°C and bacteriostatic rings were analyzed. The MICs of antibiotics were measured by the microdilution assay using 8–0.016 × MIC of untreated MRSA as described (Lepe et al., 2014).

### Quantification of persister survival

*S. aureus* treated with plasma or plasma-activated saline was diluted six times with MH medium containing antibiotics. The final antibiotic concentrations, corresponding to 10 × the minimum inhibitory concentration (MIC), were as follows: tetracycline, 500 μg/ml (MP Biomedicals); gentamycin, 200 μg/ml (Sigma); clindamycin, 5,000 μg/ml (TCI); chloramphenicol, 500 μg/ml (MP Biomedicals); ciprofloxacin, 50 μg/ml (Sigma); rifampicin, 4 μg/ml (Sigma); and vancomycin, 50 μg/ml (Sigma). The numbers of surviving bacteria were determined at the time points indicated by harvesting 100 μl of bacterial culture, which was centrifuged at 10,000 × g for 1 min. The resulting pellet was resuspended in 100 μl 0.9% NaCl. Samples were serially diluted, and 10 μl of each dilution was spotted onto MH agar plates and incubated overnight at 37°C and the numbers of surviving bacteria were determined by counting the resulting CFUs.

### Detection of reactive species in MRSA

The probes 3′-(p-aminophenyl) fluorescein (APF, Sigma), 3′-(p-hydroxyphenyl) fluorescein (HPF, Sigma), and MitoSOX™ Red mitochondrial superoxide indicator (ThermoFisher) were incubated with *S. aureus* cultures, at a final concentration of 5 μM, and trans-1-(2′-methoxyvinyl)pyrene (tMVP, J&K Scientific) was used at a final concentration of 10 μM at 37°C for 30 min. Cultures were collected by centrifugation, washed with saline (0.9% NaCl) three times and resuspended in saline at an OD_600_ of 2.0. *S. aureus* suspensions were untreated, treated with plasma directly for 40 s, or the suspensions were centrifuged and resuspended in plasma-activated saline (saline treated with plasma for 40 s). Immediately after the treatments, the fluorescence intensities were detected using a microplate reader (Thermo Scientific Varioskan Flash) at the excitation and emission wavelengths [APF and HPF: 490/515 nm; superoxide indicator: 510/580 nm; trans-1-(2′-methoxyvinyl)pyrene: 405/460 nm] of each probe.

## Results

### Plasma-induced aqueous ROS/RNS

MRSA or saline treated with the plasma device was shown in Figure 1A. Gaseous ROS and RNS were generated by the surface discharge, and some of them would diffuse across the air gap and then dissolved into the liquids. The plasma-induced aqueous ROS and RNS dissolved into saline were measured after the plasma treatment for 30 s and 40 s. Long-lived species H_2_O_2_, ${\text{NO}}_{2}^{-}$, and ${\text{NO}}_{3}^{-}$, as well as the short-lived species ^•^OH, ^1^O_2_, ^•^NO, and ONOO^−^, were detected in the saline. The concentrations of aqueous H_2_O_2_, ${\text{NO}}_{2}^{-}$, and ${\text{NO}}_{3}^{-}$ after the plasma treatment for 40 s were 23, 14, and 102 μM, respectively (Figure 1B). Electron spin resonance (ESR) spectroscopy was used for the measurement of short-lived species, in which the results were the concentrations of spin adducts, only reflecting the relative concentrations of the specific ROS and/or RNS. The concentrations of spin adducts DMPO-OH, TEMPO, Nitrocyl-Fe, and TEMPONE after the plasma treatment for 40 s were 0.1, 69, 21, and 37 μM (Figure 1B). The spin adducts concentrations after the plasma treatment for 30 s were lower, such as TEMPO was 30% lower than that after the plasma treatment for 40 s. These results suggested that the concentration of aqueous ^•^OH was very low, and the concentration of aqueous ^1^O_2_ should be much larger. The plasma generated many species ROS and RNS, which were thought to play a crucial role in the biological effects.

### Sublethal treatment with plasma increased the sensitivity of MRSA to antibiotics

The sublethal dose (reduction about 50% viability of MRSA) of plasma and plasma-activated saline were determined by exposing the bacteria to plasma for increasing times or incubating with saline treated with plasma for increasing times and subsequently determining bacterial viability. The treatment of plasma for 40 s or the saline activated by plasma for 40 s lead to about 50% death of MRSA cells (Figure S1). For safety, a lower dose was also employed, so a slightly lower dose (plasma treatment for 30 s) that lead to about 40% death of MRSA cells and the LD_50_ dose (plasma treatment for 40 s) were both used to detect the effects on antibiotic sensitivity.

**Figure 2:**
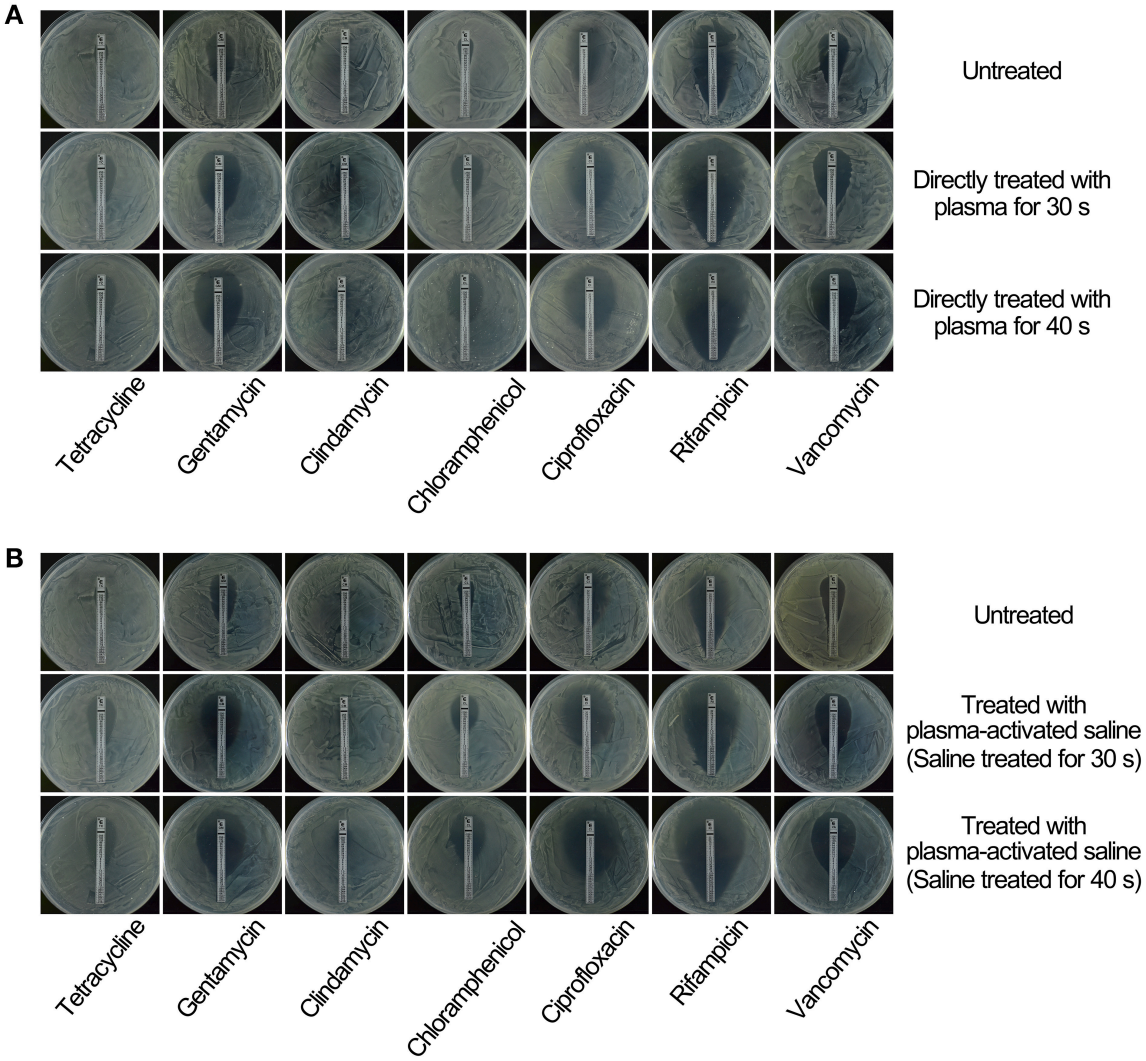
Treatment with sublethal doses of plasma or plasma-activated saline increased the sensitivity of MRSA to antibiotics. **(A)** Direct plasma treatment. **(B)** Plasma-activated saline treatment. Plasma treated, plasma-activated saline treated and untreated MRSA were plated on MH agar with Etest strips and cultured at 37°C overnight.

To study the effect of plasma on the sensitivity of MRSA to the antibiotics, MRSA were sublethally pre-treated with plasma or plasma-activated saline followed by incubation with a range of antibiotics including tetracycline, gentamycin, clindamycin, chloramphenicol, ciprofloxacin, rifampicin, and vancomycin. The minimal inhibitory concentration (MIC) of tetracycline for untreated MRSA measured by Etest paper was ~32 μg/ml, whilst the MIC of tetracycline for MRSA treated with plasma for 30 or 40 s was 10-fold lower, at ~3 μg/ml (Figure 2A). In the second approach, the MICs were measured by the microdilution assay, and the MIC of tetracycline against MRSA treated with plasma for 40 s was 8-fold lower than that against untreated MRSA (Table 1). The effects of plasma pre-treatment on MRSA sensitivity to antibiotics were independent of the particular antibiotic used, and the increase in sensitivity was observed for most of the antibiotics tested. The MICs of gentamycin against untreated MRSA and MRSA treated with plasma for 30 and 40 s measured by Etest paper were ~1.5, 1.0, and 0.5 μg/ml, respectively, and the MIC of gentamycin against MRSA treated with plasma for 40 s was 16-fold lower than that against untreated MRSA as indicated by the microdilution assay (Figure 2A; Table 1). The MICs of chloramphenicol, ciprofloxacin, rifampicin, and vancomycin against MRSA treated with plasma also decreased in varying degrees (Figure 2A; Table 1). Plasma-treated MRSA did not appear to be susceptible to treatment with clindamycin at the tested concentration (Figure 2A). Two possible explanations for this were that either the plasma treatment did not change the antibiotic sensitivity of MRSA to clindamycin or the MIC of clindamycin against MRSA was beyond the range of the Etest. Thus, MRSA susceptibility to clindamycin was measured at a higher antibiotic dose using the Kirby-Bauer method. The diameter of the bacteriostatic ring of untreated MRSA to 2,000 μg clindamycin was about 16 mm, and that of the MRSA treated for 30 and 40 s were 23 and 26 mm, respectively (Figures S2A,B). The microdilution assay also demonstrated that the MICs of clindamycin against MRSA treated with plasma for 30 and 40 s was 2- and 4-fold lower, respectively, than that against untreated MRSA (Table 1). These data indicated that sublethal plasma treatment to MRSA restored the sensitivity to five different classes of antibiotics.

**Table 1:** Antibiotic susceptibilities of *Staphylococcus aureus* treated with plasma and plasma-activated saline.

**Antibiotics**	**MIC^a^ (mg/liter) of indicated antibiotic**				
	**Untreated**	**Directly treated with plasma for 30 s**	**Directly treated with plasma for 40 s**	**Treated with plasma-activated saline (Saline treated for 30 s)**	**Treated with plasma-activated saline (Saline treated for 40 s)**
Tetracycline	50	12.5	6.25	12.5	6.25
Gentamycin	20	2.5	1.25	1.25	1.25
Clindamycin	500	250	125	250	250
Chloramphenicol	50	25	12.5	25	25
Ciprofloxacin	5	1.25	0.31	1.25	0.625
Rifampicin	0.4	0.025	0.0125	0.025	0.0125
Vancomycin	5	5	2.5	5	2.5

a *MICs were determined by broth microdilution method. The results were read after 24 h of incubation at 37°C*.

Next, the effects of plasma-activated saline upon changing the sensitivity of MRSA to these antibiotics were detected. The minimal inhibitory concentration (MIC) of tetracycline against untreated MRSA was ~32 μg/ml, whilst that for MRSA treated with saline activated by plasma for 30 or 40 s was 10-fold lower, at ~3 μg/ml (Figure 2B). The MIC of tetracycline against MRSA treated with saline activated by plasma for 40 s was also 8-fold lower than that against untreated MRSA as indicated by the microdilution assay (Table 1). The MICs of gentamycin, clindamycin, chloramphenicol, ciprofloxacin, rifampicin, and vancomycin against MRSA treated with plasma-activated saline were also lower than those against the untreated MRSA, demonstrating that plasma-activated saline exhibited the same effect in increasing the sensitivity to five different classes of antibiotics (Figures 2B, Figures S2C,D; Table 1).

### Plasma pre-treatment promoted eradication of MRSA persisters

**Figure 3:**
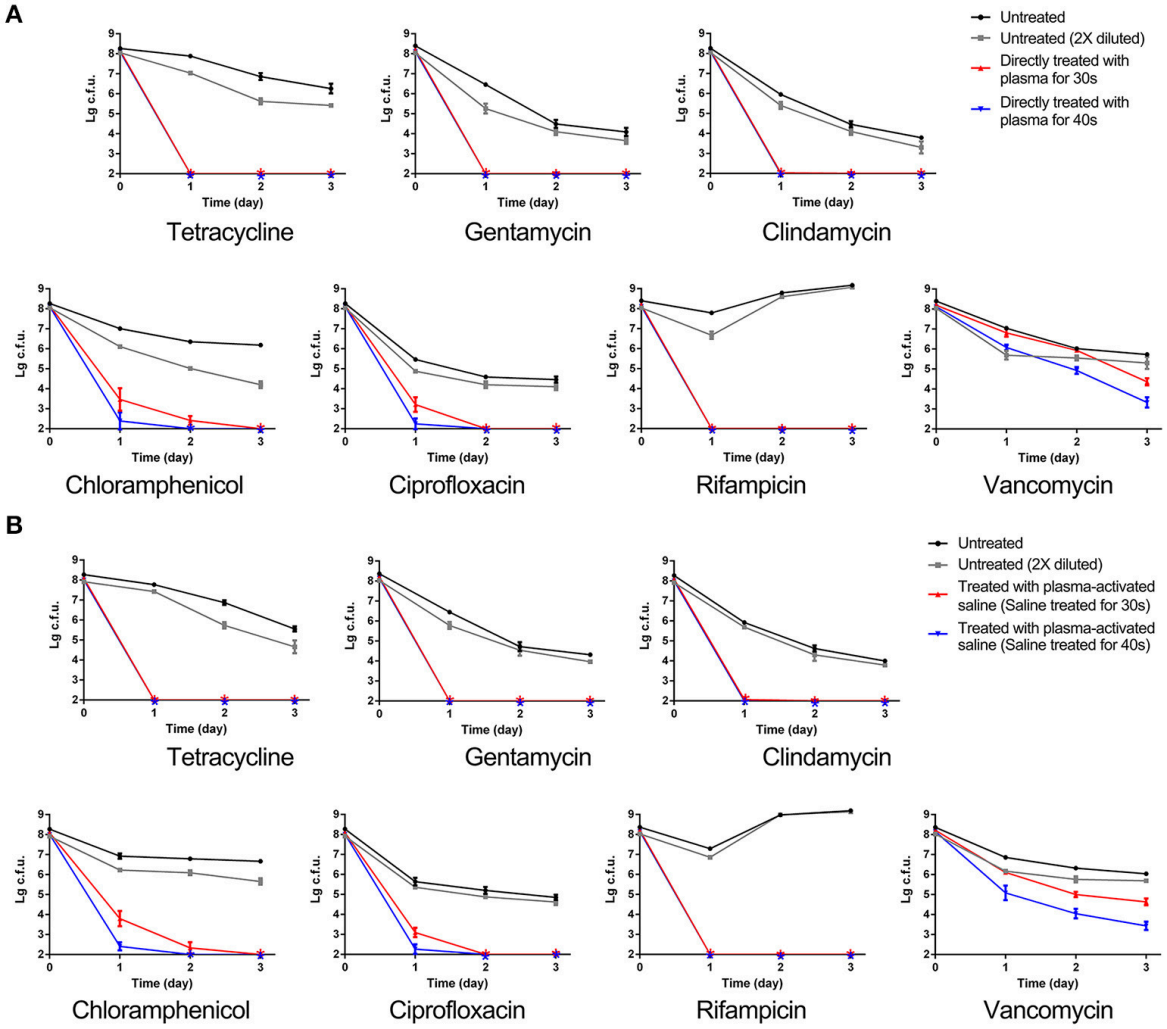
MRSA sublethally treated with plasma or plasma-activated saline increased the eradication of persisters by antibiotics. **(A)** Direct plasma treatment. **(B)** Plasma-activated saline treatment. Plasma treated, plasma-activated saline treated and untreated MRSA were grown in MHB with 10 × MIC of the indicated antibiotics, and aliquots of the cultures were taken at the times indicated, serially diluted and plated. Then the plates were cultured at 37°C overnight and the numbers of bacteria were counted. The asterisks represented eradication to the limit of detection.

Given that pre-treatment of MRSA with plasma or plasma-activated saline decreased the MIC of multiple antibiotics, we next asked whether either of these pre-treatment conditions could also promote the killing of persisters. To test this, untreated and sublethal dose plasma-treated MRSA were incubated with different antibiotics, as indicated (Figure 3A). Incubation of untreated and double-diluted untreated MRSA with the different antibiotics generally exhibited typical killing curves, with bacterial numbers decreasing from 2 × 10^8^ to 10^4^-10^6^ CFUs, with a small fraction of persisters detected after 3 days of antibiotic exposure. Of note, the numbers of untreated MRSA and double-diluted untreated MRSA that incubated with rifampicin increased after 1 day. These results indicated that the decrease of the initial MRSA numbers did not greatly influence the killing curves of antibiotics. In contrast, plasma-treatment reduced the number of MRSA persisters to below the limit of detection when incubated with tetracycline, gentamycin, clindamycin, chloramphenicol, and rifampicin within only 1 day of exposure (Figure 3A). A similar decrease was also observed within 2 and 3 days of incubation with ciprofloxacin and chloramphenicol, respectively (Figure 3A). For vancomycin, plasma treatment for 30 and 40 s effectively reduced the numbers of remaining MRSA recovered during exposure to this antibiotic to 10^4^ and 10^3^ CFUs, respectively, though these did not drop below the detection limit (Figure 3A). Treatment with plasma-activated saline exhibited the same effect of inactivating persisters as observed by direct plasma treatment (Figure 3B). The numbers of plasma-activated saline treated MRSA recovered upon exposure to antibiotics tetracycline, gentamycin, clindamycin, and rifampicin dropped to below the detection limit, but plasma-activated saline similarly only decreased the numbers of MRSA to 10^3^ CFUs upon exposure to vancomycin (Figure 3B). These results indicated that both plasma and plasma-activated saline treatment promoted the eradication of MRSA persisters by antibiotics.

### Reactive species in MRSA

**Figure 4:**
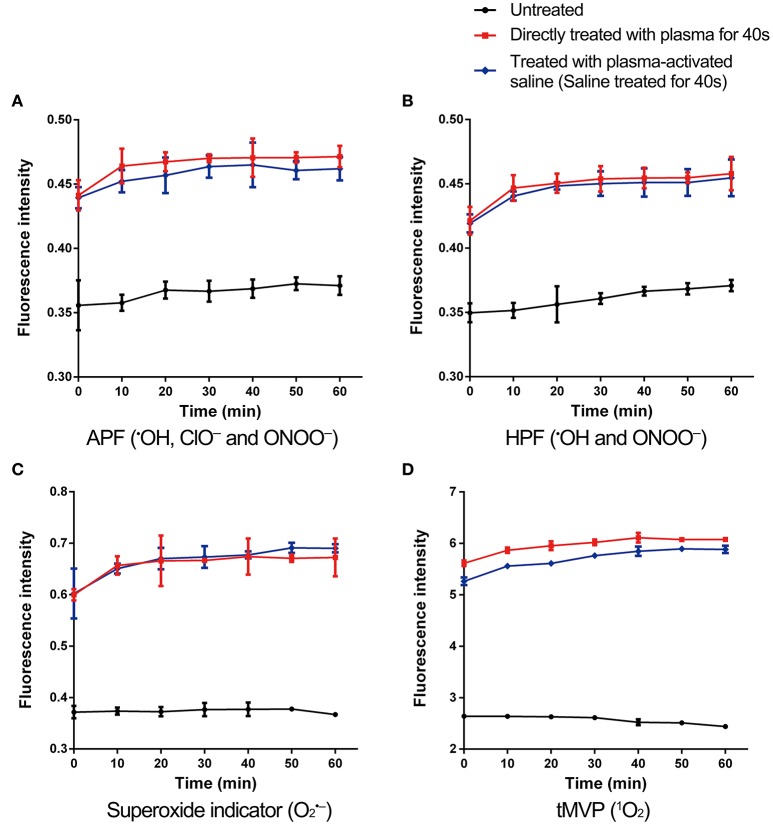
ROS and RNS levels increased in plasma-treated and plasma-activated saline-treated MRSA. MRSA incorporated with APF **(A)**, HPF **(B)**, superoxide indicator **(C)**, and tMVP **(D)**, which measured the ROS or RNS as indicated, treated with plasma for 40 s or saline treated with plasma for 40 s. Then the fluorescence intensities of were measured in plasma-treated, plasma-activated saline-treated, and untreated MRSA. Data are representative of three independent experiments. Error bars represent s.d.

Plasma-treated solutions contain many active species, such as the long-lived species H_2_O_2_, ${\text{NO}}_{2}^{-}$, and ${\text{NO}}_{3}^{-}$ as well as the short-lived species ^•^OH, ^1^O_2_, ^•^NO, ${\text{O}}_{2}^{•-}$, and ONOO^−^ (Figure 1B). To evaluate the impact of long-lived species, a mixture of H_2_O_2_ (500 μM), ${\text{NO}}_{2}^{-}$ (250 μM), and ${\text{NO}}_{3}^{-}$ (750 μM) was used to treat the MRSA. H_2_O_2_, ${\text{NO}}_{2}^{-}$, and ${\text{NO}}_{3}^{-}$ treatment did not change the antibiotic susceptibility of MRSA and the inactivation of persisters by antibiotics, indicating that the three long-lived species are not likely the direct and driving factor (Figure S3). Hence, short-lived species were considered more important, and the ROS and RNS in MRSA cells were measured. MRSA were incubated with 3′-(p-aminophenyl) fluorescein (APF) for ^•^OH, ClO^−^, and ONOO^−^, HPF for ^•^OH and ONOO^−^, MitoSOX™ Red mitochondrial superoxide indicator for ${\text{O}}_{2}^{•-}$ and trans-1-(2′-methoxyvinyl)pyrene (tMVP) for ^1^O_2_, then exposed to plasma or plasma-activated saline for 40 s, and the levels of different ROS/RNS determined by changes in the fluorescence intensities (Figure 4). After plasma treatment, the fluorescence intensities of the four probes increased. The fluorescence intensities of MRSA incubated with APF and HPF increased slightly after 30 min of plasma treatment, by 28 and 25%, respectively, which indicates an increase in the levels of ^•^OH, ClO^−^, and ONOO^−^ (Figures 4A,B). Fluorescence of MRSA incubated with MitoSOX™ Red mitochondrial superoxide indicator increased by 77%, and the trans-1-(2′-methoxyvinyl)pyrene (tMVP) for ^1^O_2_ increased by 130% after 30 min of plasma treatment (Figures 4C,D). The fluorescence intensities of the probes which incubated with plasma-activated saline-treated MRSA were slightly weaker than those of the direct plasma-treated samples. These data suggested that the plasma and plasma-activated saline treatment induced the increases of many species of ROS and RNS in MRSA cells.

## Discussion

In this study, we demonstrated that treating MRSA sublethally with plasma-generated ROS and RNS decreased the MICs of several antibiotics and increased persister eradication, along with increases in the levels of ROS and RNS in MRSA cells. Plasma-activated saline had the same effect upon the antibiotic sensitivity of MRSA and persister inactivation as direct plasma treatment, suggesting that the plasma-generated ROS and RNS could be applied in both gaseous or aqueous form depending on the mode of application. The short-lived species in the plasma-activated saline had short half-lives, but they could react and generate long-lived species, which also could generate to short-lived species reversibly, such as ONOO^−^ generated from ${\text{NO}}_{3}^{-}$ (Oehmigen et al., 2011; Liu et al., 2017). Plasma generated various ROS, which are involved in a great many chemical reactions (Oehmigen et al., 2011). The underlying reactions and detailed mechanisms of plasma-activated saline are still not well understand and require further study.

Unlike other treatments that have previously been used as antibiotic adjuvants to enhance ROS production in bacterial cells, plasma induces the production of a wider range of ROS species (Morones-Ramirez et al., 2013; Shen et al., 2016). The ROS and RNS generated by the plasma constitute a complex mix of products, including ^•^OH, ^1^O_2_, ${\text{O}}_{2}^{•-}$, ONOO^−^, and ClO^−^ (Liu et al., 2016). Subsequently, these many different ROS or RNS species were also detected in plasma-treated bacterial cells. The intracellular ROS and RNS could induce oxidative stresses, such as damaging lipids, proteins and DNA by ^•^OH, ^1^O_2_, and ${\text{O}}_{2}^{•-}$, as well as protein damages by ONOO^−^ and ClO^−^ (Davies, 2016). ${\text{O}}_{2}^{•-}$ could be detoxified by endogenous antioxidants of the oxidative response, but no enzyme can detoxify ^•^OH or ^1^O_2_, and MRSA could not detoxify all the multiple reactive species induced by plasma (Vatansever et al., 2013). It was speculated that the compound damages stimulated multiple response pathways and kept MRSA busy with repairing, which contributed to the effects of antibiotics.

Reactive species generated by plasma could also induce damages in eukaryotic cells, subsequently, the safety and toxicity of this application should be considered. The CC_50_ of the plasma treatment used in this study for human primary dermal fibroblasts was about 40 s treatment, which was close to the LD_50_ on MRSA (Figure S4A). Comparing with bacterial cells, the cultured cells are more easily to be inactivated *in vitro* because of the lack of cell wall. However, the plasma treatment did not increase micronuclei formation in fibroblast cells (Figures S4B,C). Coincidently, a newly published paper found similar results on lymphocyte TK6 cells (Bekeschus et al., 2018). Further, the olive tail moment of plasma-treated cells exhibited little difference with that of untreated cells (Figures S4D,E). So the plasma treatment did not remarkably increase the mutagenicity of fibroblasts as demonstrated by both micronucleus assay and comet assay. Besides, Maisch et al. (2012) showed that gas plasma could efficiently inactivate *S. aureus* and *Escherichia coli* on pig skin without inducing morphological changes or damage-related apoptosis. Clinical trials also demonstrated that 5 min daily treatment with plasma decreased bacteria in chronic wounds of patients without side effects (Isbary et al., 2010). These studies demonstrated the safety of plasma treatment under limited conditions. Many topical biocides are toxic, and the plasma could be developed as alternative, especially the plasma-activated saline could be the save alternative (Wales and Davies, 2015). When used as a sensitizer with antibiotics as was done in this study, the doses of plasma were much lower than that used for bacteria inactivation, which would reduce the risk of toxicity and improve the safety.

In conclusion, sublethal treatment with plasma-generated ROS and RNS decreased the MICs of several antibiotics and increased persister eradication, along with increases in the levels of ROS and RNS in MRSA. Plasma and plasma-activated saline could be explored as a novel antibiotic sensitizer to generate oxidative stress to combat the increasing problem of antibiotic resistance. Further studies are needed to test these methods against other multidrug-resistant bacteria and to elucidate the underlying mechanism.

## Author contributions

LG, RX, and YZ designed and executed most of the experiments and analyzed the data. ZL assisted in the execution of some experiments. LG, DL, and MK wrote the manuscript. MK, LG, DL, XW, and HC reviewed and approved the final version of the manuscript.

### Conflict of interest statement

The authors declare that the research was conducted in the absence of any commercial or financial relationships that could be construed as a potential conflict of interest.
