# Supplementary material for: Gas Plasma Pre-treatment Increases Antibiotic Sensitivity and Persister Eradication in Methicillin-Resistant Staphylococcus aureus
Source: Front Microbiol. 2018 Mar 23;9:537. doi: 10.3389/fmicb.2018.00537 (PMC5876240; doi:10.3389/fmicb.2018.00537)

SUPPLEMENTARY MATERIAL

Materials and Methods

Cell viability assay

Human primary dermal fibroblasts (ATCC PCS-201-012) were cultured using Fibroblast Basal Medium (ATCC PCS-201-030) supplemented with Fibroblast Growth Kit-Low serum (ATCC PCS-201-041) at 37°C with 5% CO_2. To measure cell viability, 5000 cells per well were seeded in 96-well plates with six replicates. One day after, cells were washed with saline once, covered with saline, and either treated with plasma directly or covered with plasma-activated saline followed by incubation for 30 min at room temperature. Then the cell viability were measured using MTT Cell Proliferation and Cytotoxicity Assay Kit (Sangon Biotech). After incubation for 2 h, the light absorbance at 570 nm was recorded by a microplate reader (Thermo Scientific). The CC_{50} were calculated using SPSS statics 23.0.

Micronucleus assay

To measure the mutagenicity of plasma, 10000 cells per well were seeded in 24-well plates with four replicates. One day after, cells were washed with saline once, covered with saline, and either treated with plasma directly or covered with plasma-activated saline followed by incubation for 30 min at room temperature. Saline were aspirated, fresh medium were added and incubated for 22 hours at 37°C with 5% CO_2. Medium were removed, fresh medium with cytochalasin B (6 μg/ml) were added and incubated for 24 hours at 37°C with 5% CO_2. Then the cells were fixed using 4% paraformaldehyde in PBS buffer for 15 min at 37°C. After fixation, the cells were incubated with 300 nM 4',6-diamidino-2-phenylindole (DAPI, Thermo-Fisher) in PBS for 10 min. The cells were imaged using EVOS FL Auto Cell Imaging System (Thermo Scientific) and 500-700 binucleated cells were analyzed.

Comet assay

Cells were grown to approximately 80% confluence before treatment, washed with saline twice, covered with saline, and either treated with plasma directly or covered

with plasma-activated saline followed by incubation for 30 min at room temperature. Treated cells were collected by trypsin/EDTA (Gibco) digestion. Then 2×10^3 HeLa cells in 20 μL were mixed with 200 μL of low melting point agarose (0.7%), and 50 μL of this mixture was added onto comet slides (Trevigen). The slides were immersed in lysis solution (2 M NaCl, 30 mM EDTA, 10 mM Tris pH 8.5, 0.1% lauroylsarcosine and 1% Triton X-100) at 4°C overnight. The cells were incubated with electrophoresis buffer (200 mM NaOH, 1 mM EDTA, pH>13) for 1 h at 4°C and subjected to electrophoresis in the same buffer at 1V/cm for 30 min. After electrophoresis, the slides were incubated in 70% ethanol for 30 min at room temperature, dried at 37°C, and stained with SYBR Gold (Thermo-Fisher) for 30 min at room temperature. Comets were imaged using EVOS FL Auto Cell Imaging System, and 150 comets were analyzed by HCS Studio 3.0 bioapplication software (Thermo Scientific).

Figure S1. Plasma or plasma-activated saline treatment reduced MRSA viability. MRSA was directly treated with plasma, or with saline that had been treated with plasma, for the indicated time periods and then incubated for 30 min at room temperature. Surviving cells were quantified by serial dilution, plating, and counting of the resulting CFUs.

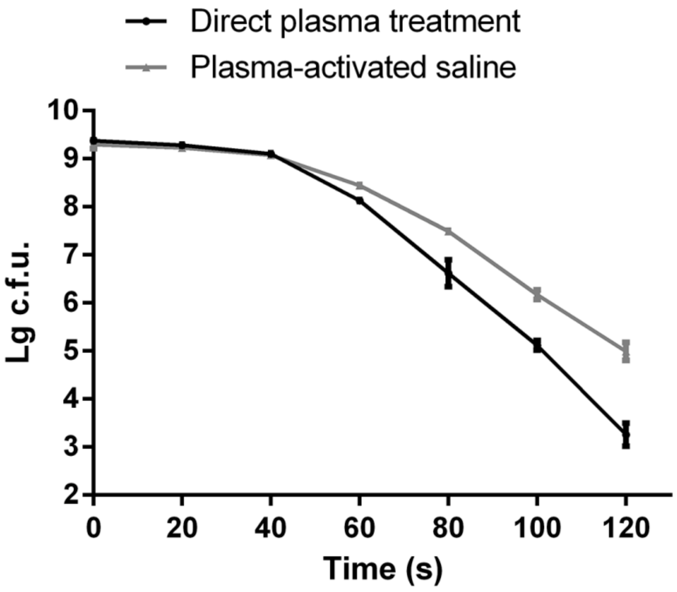

Figure S2. Analysis of the sensitivity to clindamycin of MRSA using the Kirby-Bauer method. **A** and **B**. Direct plasma treatment. **C** and **D**. Plasma-activated saline treatment. A and C. The bacteriostatic rings of clindamycin. The treated and untreated MRSA were plated on MH agar with clindamycin paper and cultured at 37°C overnight. B and D. Statistical analysis of bacteriostatic rings. ***, $p \leq 0.001$; **, $p < 0.01$.

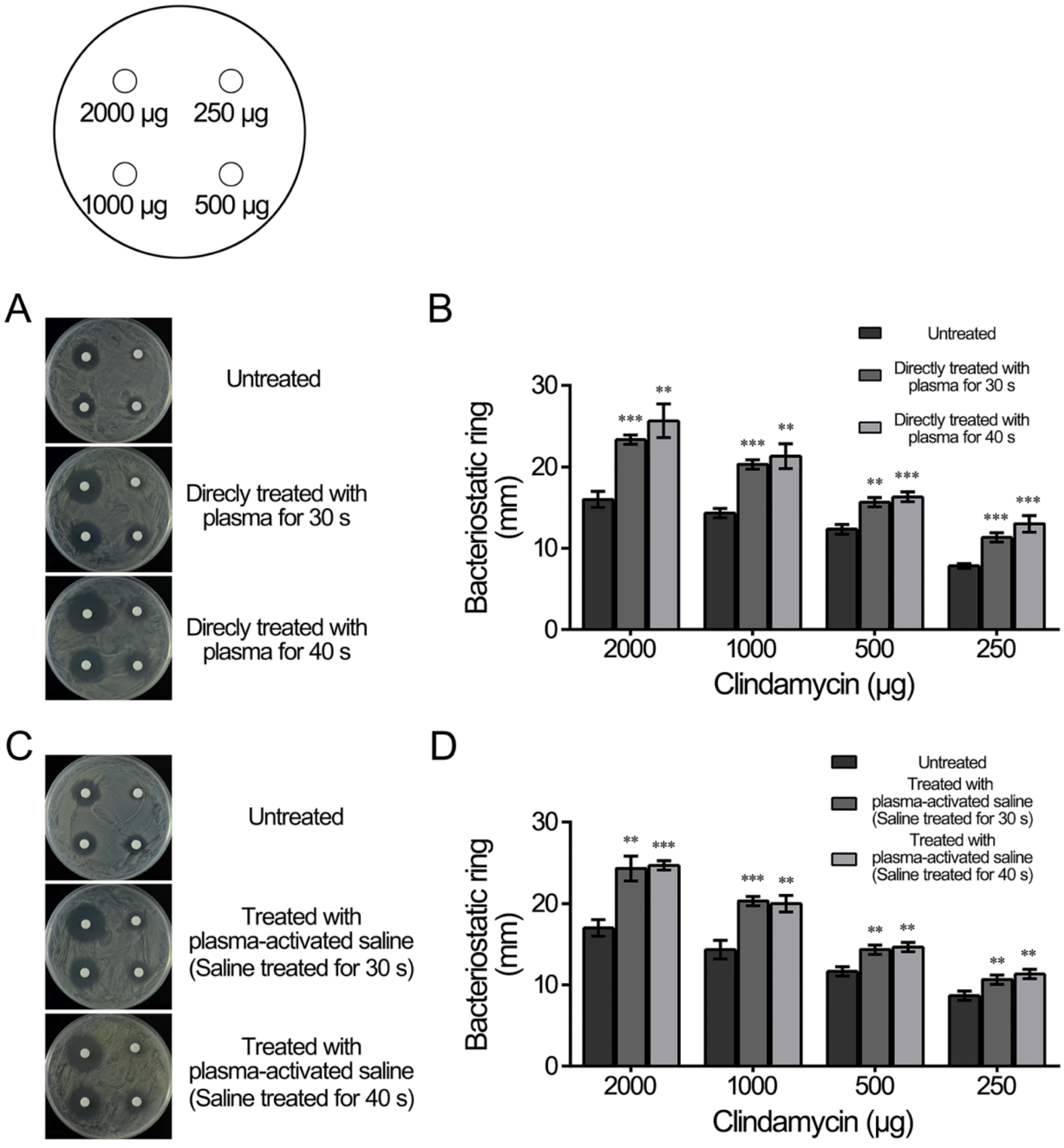

Figure S3. Treatment with H_2O_2, NO_2^-, and NO_3^- at room temperature for 30 min did not change the antibiotic sensitivity of MRSA nor persister eradication. **A**. Etest analysis of the antibiotics sensitivities of H_2O_2, NO_2^- and NO_3^--treated and untreated MRSA. MRSA treated with 250 μM H_2O_2 + 125 μM NO_2^- + 375 μM NO_3^- or 500 μM H_2O_2 + 250 μM NO_2^- + 750 μM NO_3^- and untreated MRSA were plated on MH agar with Etest strips and cultured at 37°C overnight. **B**. Persister analysis of H_2O_2, NO_2^- and NO_3^--treated and untreated MRSA. MRSA treated with 250 μM H_2O_2 + 125 μM NO_2^- + 375 μM NO_3^- or 500 μM H_2O_2 + 250 μM NO_2^- + 750 μM NO_3^- and untreated MRSA were grown in MHB with 10×MIC of indicated antibiotics, and aliquots of the cultures were taken at the times indicated, serially diluted and plated. Then the plates were cultured at 37°C overnight and the numbers of bacteria were counted.

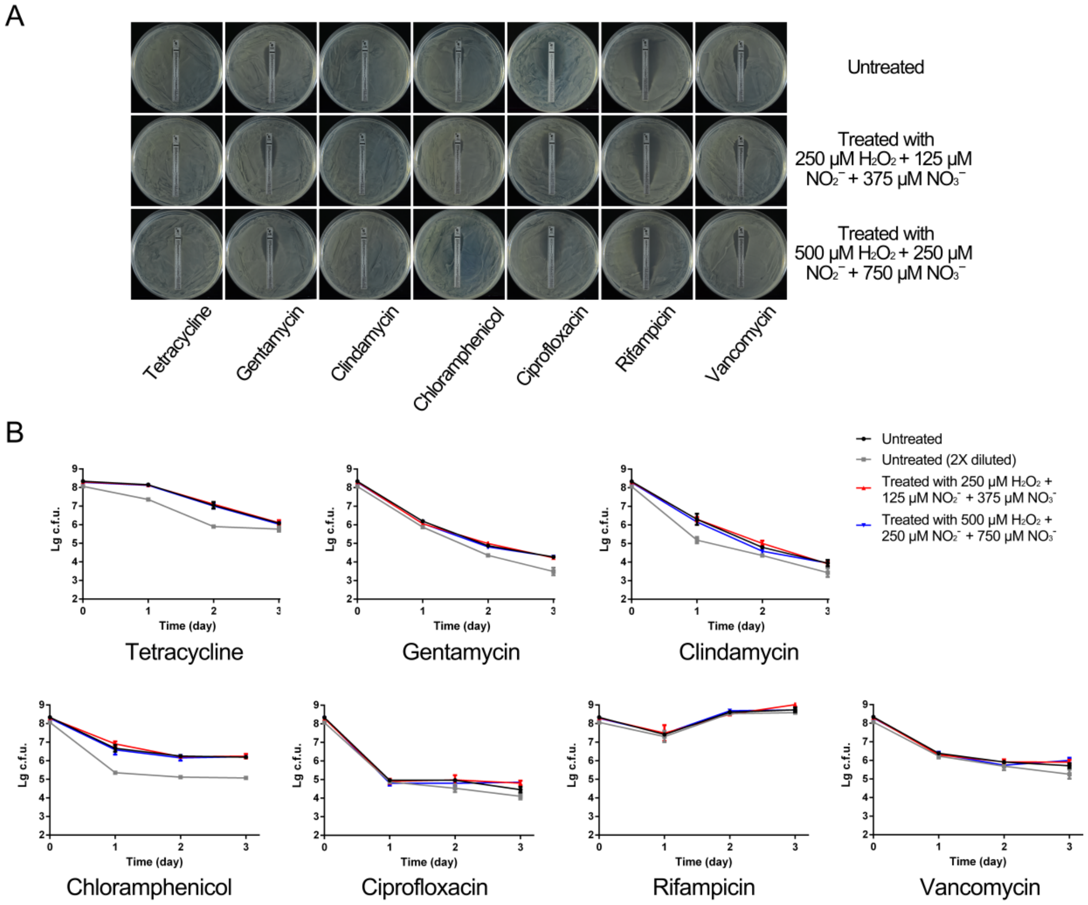

Figure S4. The toxicity and mutagenicity of plasma treatment on human primary dermal fibroblasts. **A**. Cell viability. Cell viability of cells treated with plasma or untreated were measured using MTT assay. **B**. Micronucleus. The micronucleus in cells treated with plasma or untreated were measured using mitomycin C treatment (50 ng/ml) as a control. The bars represent 50 μm. **C**. Quantitative analysis of micronucleus. **D**. Comet. Cells treated with plasma or untreated were subjected to single-cell gel electrophoresis. The bars represent 100 μm. **E**. Quantitative analysis of olive tail moment of comets.

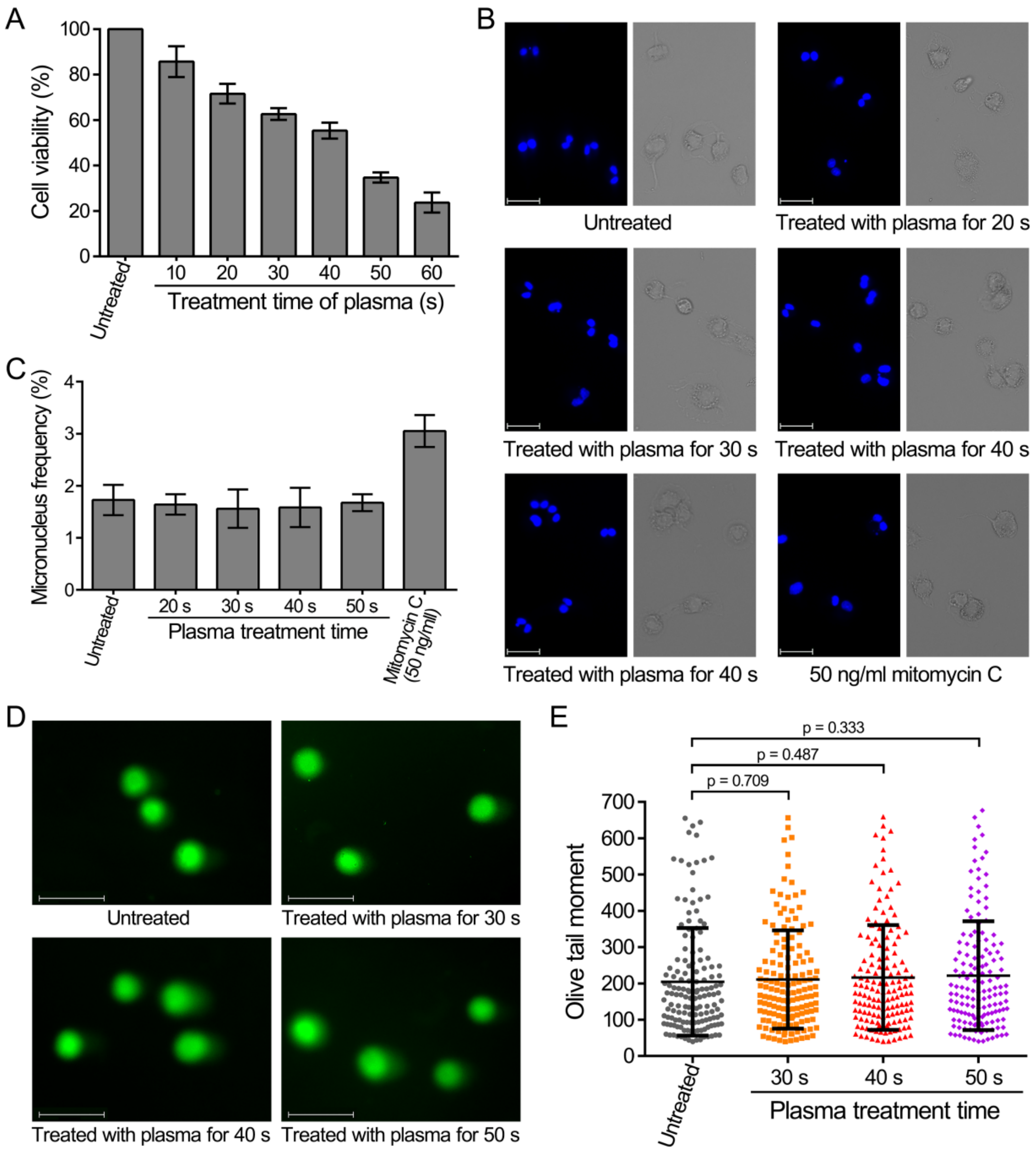

Supplement: Supplementary file 1 [file Presentation1.PDF]
